# Nitrofurantoin as a preferred first-line therapy for urinary tract infection: A comparison using urinary tract infection-specific antibiograms

**DOI:** 10.14440/bladder.0018

**Published:** 2025-09-04

**Authors:** Colby P. Souders, Andrew Clark, Bonnie C. Prokesch, Philippe E. Zimmern

**Affiliations:** 1 Department of Urology, School of Medicine, University of Kansas Medical Center, Kansas City, Kansas 66160, United States of America; 2 Department of Pathology, School of Medicine, University of Texas Southwestern Medical Center, Dallas, Texas 75390, United States of America; 3 Department of Infectious Disease, School of Medicine, University of Texas Southwestern Medical Center, Dallas, Texas 75390, United States of America; 4 Department of Urology, School of Medicine, University of Texas Southwestern Medical Center, Dallas, Texas 75390, United States of America

**Keywords:** Nitrofurantoin, Antibiogram, Empiric therapy, Urinary tract infection

## Abstract

**Background:**

Local antibiograms are a highly useful tool to guide empiric antibiotic therapy for a variety of infections.

**Objectives:**

This study compares nitrofurantoin (NF) susceptibility in outpatient urine isolates using antibiogram data from two United States academic medical centers.

**Methods:**

In this brief report, we compare antibiograms of urinary isolates from two distinct academic institutions—the University of Texas Southwestern (UTSW) in Dallas, Texas, and the University of Kansas Medical Center (KU) in Kansas City, Kansas—focusing on the most common uropathogens susceptible to NF to evaluate whether NF remains an appropriate first-line agent for uncomplicated cystitis.

**Results:**

For the 2022 antibiogram, KU tested 5,524 urinary isolates and UTSW tested 2,530 urinary isolates. The susceptibility data were consistent across the two institutions, suggesting that NF can continue to be considered a first-line therapy for uncomplicated urinary tract infections in women.

**Conclusion:**

This report highlights the importance of clinicians consulting their local antibiograms to inform empiric antibiotic therapy.

## 1. Introduction

Nitrofurantoin (NF) is ideally suited for the treatment of uncomplicated urinary tract infections (UTIs) in women, owing to its favorable safety profile and its ability to achieve bactericidal concentrations in urine but not in plasma.^1^ Furthermore, its exclusive use in the treatment of cystitis results in fewer “off-target” effects on fecal and vaginal microbiomes.^2^ In addition, resistance to NF is more difficult for uropathogens to develop compared with other antimicrobial agents, owing to its unique mechanism of action.^3^,^4^ NF’s bactericidal activity involves multiple sites of action, including inhibition of ribosomal translocation, damage to bacterial DNA, and inhibition of protein synthesis.^5^ Because this antibiotic employs multiple mechanisms to exert its activity, resistance remains uncommon. Given that NF has been in clinical use since the 1950s,^4^ and in the context of rising antimicrobial resistance, concerns have emerged regarding its continued suitability as a first-line agent for uncomplicated UTIs. To address this concern, we compared the rates of NF susceptibility among patient populations at two different academic medical centers in the United States to evaluate whether NF remains an appropriate first-line treatment for uncomplicated cystitis.

## 2. Patients and methods

Outpatient urinary isolates of the main uropathogens inherently susceptible to NF, along with their susceptibilities to NF, were compared using antibiograms from the year 2022 provided by the microbiology laboratories of two tertiary institutions: University of Texas Southwestern (UTSW) in Dallas, Texas, and University of Kansas Medical Center (KU) in Kansas City, Kansas. Organisms not treatable with NF (e.g., *Pseudomonas*) were excluded. At both institutions, after bacterial growth was observed on plates prepared from urine specimens submitted for culture, technicians identified the predominant colonies with significant growth (typically >50 colony-forming units). Matrix-assisted laser desorption/ionization time-of-flight mass spectrometry was then used for bacterial identification. The isolates were subsequently tested for antimicrobial susceptibility using a Microscan machine, which determines susceptibilities according to Clinical and Laboratory Standards Institute criteria to generate minimum inhibitory concentration reports. These reports were then aggregated to create antibiograms, which summarize antibiotic susceptibilities of bacterial pathogens at each institution. Additional clinical data, including site of urine collection (urology clinic versus other outpatient clinic), patient age, and patient gender, were also analyzed.

## 3. Results and discussion

For the antibiogram from the year 2022, KU tested 5,524 total urinary isolates, including *Escherichia coli* (*n* = 3,496; 98% susceptible to NF), *Klebsiella oxytoca* (*n* = 157; 90% susceptible), *Enterococcus faecalis* (*n* = 664; 99% susceptible), and *Citrobacter freundii* (*n* = 95; 97% susceptible). NF provided less satisfactory coverage for *Enterobacter cloacae* species complex (21% susceptible), *Klebsiella aerogenes* (11% susceptible), and *Klebsiella pneumoniae* species complex (44% susceptible) (Table 1).

**Table 1:** 2022 antibiogram of outpatient urine isolates

2022 antibiogram of outpatient urine isolates	Nitrofurantoin			

	KU		UTSW	
	
	n	%S	n	%S
Enterobacterales (Gram-negative bacteria)				
*Citrobacter freundii* complex	95	97	73	93
*Citrobacter koseri*	83	51	76	66
*Enterobacter cloacae* complex	121	21	121	26
*Escherichia coli*	3496	98	1311	96
*Klebsiella aerogenes*	83	11	77	21
*Klebsiella oxytoca/Raoultella ornithinolytica*	157	90	97	91
*Klebsiella pneumonia* complex	777	44	372	42
Gram-positive cocci				
*Enterococcus faecalis*	664	99	403	99

Notes: This table compares outpatient uropathogens and their susceptibilities to nitrofurantoin at two academic medical centers. “%S” denotes the nitrofurantoin susceptibility rate.

Abbreviations: KU: University of Kansas Medical Center; UTSW: University of Texas Southwestern.

At UTSW, 2,530 total urinary isolates were tested, including *E. coli* (*n* = 1,311; 96% susceptible), *K. oxytoca* (*n* = 97; 91% susceptible), *E. faecalis* (*n* = 403; 99% susceptible), and *C. freundii* (*n* = 73; 93% susceptible). Consistent with KU antibiogram data, NF provided less satisfactory coverage for *E. cloacae* species complex (26% susceptible), *K. aerogenes* (21% susceptible), and *K. pneumoniae* species complex (42% susceptible) (Tables 1 and 2).

**Table 2:** Nitrofurantoin susceptibilities of outpatient urine isolates for predominant Gram-positive and Gram-negative uropathogens (UTSW 2022 antibiogram)

Nitrofurantoin susceptibility of outpatient urine isolate (UTSW 2022 antibiogram)		*Escherichia coli*				*Enterococcus faecalis*			
	
		Male		Female		Male		Female	
				
Patient age	Clinic location	*n*	%S	*n*	%S	*n*	%S	*n*	%S
<40	General outpatient clinic	2	100	168	98	2	100	28	100
	Urology clinic	8	100	50	94	8	100	31	100
40–60	General outpatient clinic	22	91	216	98	9	100	19	100
	Urology clinic	36	83	122	93	36	100	80	100
>60	General outpatient clinic	90	93	613	97	69	100	119	99
	Urology clinic	116	99	406	94	242	100	199	100
Total		274	95	1575	96	366	100	476	100

Notes: This table presents nitrofurantoin susceptibilities of predominant Gram-positive and Gram-negative uropathogens from different outpatient care sites. “%S” denotes the nitrofurantoin susceptibility rate.

Abbreviation: UTSW: University of Texas Southwestern.

At UTSW, NF susceptibilities did not differ across patient age, clinic location, or patient gender (Table 2).

This study adds to the existing literature supporting the continued use of NF as a first-line agent for uncomplicated UTIs. In an era of rising antibiotic resistance, our findings demonstrate that many strains of *E. coli*—the most common uropathogen worldwide—remain susceptible to NF.^5^

Although this study shows that NF susceptibilities remain comparable between two institutions in different regions of the United States, there are notable limitations. Although both institutions represent relatively large patient populations, the inclusion of only two sites may limit generalizability. In addition, variables such as comorbidities, prior antibiotic exposure, and recurrent UTI status were not controlled or addressed in this brief report. These factors substantially influence pathogen resistance and antibiotic selection; however, the present study was designed to assess cumulative susceptibility data for NF-susceptible organisms at a broader population level.

## 4. Conclusion

Local antibiograms for UTIs are valuable tools to guide empiric antibiotic therapy. This brief report highlights that antibiograms for the most common uropathogens are consistently favorable for the use of NF—particularly for *E. coli*, *K. oxytoca, E. faecalis*, and *C. freundii*—regardless of clinic location, patient gender, or institution. As suggested by current guidelines^6^ and reinforced by these data, which were consistent across two institutions with distinct patient populations in different regions of the United States, NF should continue to be considered a primary first-line agent for uncomplicated UTIs caused by the most common uropathogens. However, further studies are warranted to confirm and generalize these findings. In addition, providers are encouraged to regularly review antibiograms at their respective institutions to aid in selecting appropriate empiric antibiotic therapy for UTIs and other infections.

## Data Availability

Data is available from the corresponding author upon reasonable request.
